# The current landscape of European registries for rare endocrine conditions

**DOI:** 10.1530/EJE-18-0861

**Published:** 2018-11-08

**Authors:** S R Ali, J Bryce, M Cools, M Korbonits, J G Beun, D Taruscio, T Danne, M Dattani, O M Dekkers, A Linglart, I Netchine, A Nordenstrom, A Patocs, L Persani, N Reisch, A Smyth, Z Sumnik, W E Visser, O Hiort, A M Pereira, S F Ahmed, on behalf of Endo-ERN

**Affiliations:** 1 Developmental Endocrinology Research Group, School of Medicine, Dentistry & Nursing, University of Glasgow, UK; 2 Office for Rare Conditions, Royal Hospital for Children & Queen Elizabeth University Hospital, Glasgow, UK; 3 Department of Internal Medicine and Paediatrics, Ghent University; 4 Department of Paediatric Endocrinology, Ghent University Hospital, Ghent, Belgium; 5 Department of Endocrinology, William Harvey Research Institute, Barts and the London School of Medicine, Queen Mary University of London, London, UK; 6 Dutch Adrenal Network (AdrenalNET), JH Soest, the Netherlands; 7 National Centre for Rare Diseases, Istituto Superiore di Sanità, Rome, Italy; 8 Diabetes Center AUF DER BULT, Hannover, Germany; 9 Genetics and Genomic Medicine Programme, UCL GOS Institute of Child Health, London, UK; 10 Departments of Medicine & Clinical Epidemiology, Leiden University Medical Centre, Leiden, the Netherlands; 11 APHP, Bicêtre Paris Sud, le Kremlin Bicêtre, France; 12 Sorbonne Université, Inserm, Centre de recherche Sainte Antoine, APHP, Hôpital des Enfants Armand Trousseau, Paris, France; 13 Pediatric Endocrinology and Inborn Errors of Metabolism, Karolinska University Hospital, Stockholm, Sweden; 14 Department of Laboratory Medicine, Clinical Genetics and Endocrinology Laboratory, Semmelweis University, Budapest, Hungary; 15 Division of Endocrine and Metabolic Diseases, Istituto Auxologico Italiano; 16 Department of Clinical Sciences and Community Health, University of Milan, Milan, Italy; 17 Medizinische Klinik und Poliklinik IV, Klinikum der Universität München, Munich, Germany; 18 Department of Pediatrics, Motol University Hospital, Prague, Czech Republic; 19 Erasmus Medical Centre, Department of Internal Medicine, Academic Centre for Thyroid Diseases, Rotterdam, the Netherlands; 20 Division of Paediatric Endocrinology and Diabetes, Department of Paediatrics and Adolescent Medicine, University of Lübeck, Lübeck, Germany; 21 Division of Endocrinology, Department of Medicine, Leiden University Medical Center, Leiden, the Netherlands

## Abstract

**Objective:**

To identify cross-border international registries for rare endocrine conditions that are led from Europe and to understand the extent of engagement with these registries within a network of reference centres (RCs) for rare endocrine conditions.

**Methods:**

Database search of international registries and a survey of RCs in the European Reference Network for rare endocrine conditions (Endo-ERN) with an overall response rate of 82%.

**Results:**

Of the 42 conditions with orphacodes currently covered within Endo-ERN, international registries exist for 32 (76%). Of 27 registries identified in the Orphanet and RD-Connect databases, Endo-ERN RCs were aware of 11 (41%). Of 21 registries identified by the RC, RD-Connect and Orphanet did not have a record of 10 (48%). Of the 29 glucose RCs, the awareness and participation rate in an international registry was highest for rare diabetes at 75 and 56% respectively. Of the 37 sex development RCs, the corresponding rates were highest for disorders of sex development at 70 and 52%. Of the 33 adrenal RCs, the rates were highest for adrenocortical tumours at 68 and 43%. Of the 43 pituitary RCs, the rates were highest for pituitary adenomas at 43 and 29%. Of the 31 genetic tumour RCs, the rates were highest for MEN1 at 26 and 9%. For the remaining conditions, awareness and participation in registries was less than 25%.

**Conclusion:**

Although there is a need to develop new registries for rare endocrine conditions, there is a more immediate need to improve the awareness and participation in existing registries.

## Introduction

Rare diseases or rare conditions are defined by the European Union (EU) as life-threatening or chronic debilitating conditions with a prevalence rate of less than 5 per 10 000 (European Commission (EC) Regulation # 141/2000). The management of these conditions requires coalesced efforts over the life time of the patient to reduce morbidity and mortality in affected individuals. Rare endocrine conditions pose a particular challenge due to current knowledge gaps regarding long-term outcome, lack of expert and evidence-based multidisciplinary care resulting in substantial variation in care. Registries have the potential to improve patient care and healthcare planning in those with rare conditions; they enable pooling of data for research activities and surveillance; by creating a virtual environment of collaboration, they can also facilitate communication between professionals and affected individuals (EURCERD core recommendations on rare disease patient registration and data collection. http://www.eucerd.eu/wpcontent/uploads/2013/06/EUCERD_Recommendations_RDRegistryDataCollectionadopted.pdf; last accessed May 25, 2018). Multicentre collaboration between centres of expertise within registries also plays a vital role in the development of clinical benchmarks, thereby acting as a platform for quality improvement.

Over 800 rare disease registries are reported to exist in Europe with some operating on an international, national or regional scale (1, 2). A requirement for international registration of patients with rare conditions is widely recognized and supported by the EU (International Rare Disease Research Consortium. http://wwwirdirc.org; last accessed May 11 2018) (3); European initiatives including Orphanet (https://www.orpha.net/consor/cgi-bin/index.php; last accessed August 1 2018) and RD-Connect (http://catalogue.rd-connect.eu/; last accessed August 1 2018) (4) have attempted to identify existing registries. The use of cross-border international registries offers clear benefits for collaboration and standardized data collection for rare conditions.

European Reference Networks (ERNs) are networks of reference centres (RCs) across Europe that aim to manage rare medical conditions that require specialist attention and a concentration of knowledge and resources (5, 6). The ERN for rare endocrine conditions (Endo-ERN) is the largest ERN with 71 RCs from 19 member states (endo-ern.eu). It currently includes 42 conditions with orphacodes that are organised into eight broad categories termed ‘Main Thematic Groups (MTGs)’ which include Adrenal Disorders, Disorders of Calcium and Phosphate Homeostasis, Genetic Disorders of Glucose and Insulin Homeostasis, Genetic Endocrine Tumour Syndromes, Growth and Genetic Obesity Syndromes, Pituitary Disorders, Sex Development and Maturation Disorders and Thyroid Disorders. It is estimated that these 71 RCs may care for over 60 000 patients with the above groups of conditions.

Although registries are regarded as essential for Endo-ERN, knowledge on the range of registries that exist for cross-border sharing of information on rare endocrine conditions in Europe is scarce. Furthermore, the extent of awareness, participation and availability of registries for the wide range of conditions within Endo-ERN is unclear. The development of Endo-ERN provided a perfect opportunity to identify registries that already exist in Europe for the rare endocrine conditions that have been included in the network and to also determine the extent of engagement in registries of health professionals within an expert network such as Endo-ERN.

## Methods

To identify the registries that currently exist for rare endocrine conditions, the investigators examined the outputs from Orphanet (https://www.orpha.net/consor/cgi-bin/index.php; last accessed August 1 2018) and RD-Connect (http://catalogue.rd-connect.eu/; last accessed August 1 2018) projects using Orphacodes for all conditions and, in addition, surveyed Endo-ERN members between October and November 2016. For the purpose of this mapping exercise, an international registry was defined as a patient registry that collected uniform information on individual patients (7) and was used by more than one country within Europe and where the coordinating centre was based in Europe. Within Endo-ERN, all 71 RC leads from 19 countries were invited to complete a survey documenting their awareness of international, national and local registries and active participation in registries at an international, national and local level. RC leads were asked to complete their responses, for each condition that they had been approved for, using preset answer categories (Yes/No). To understand future priorities, respondents were asked to denote 1–5 on a Likert scale to assess the level of priority for need of a registry for a given condition, where 5 was the greatest priority.

### Statistical analysis

Categorical data were analysed using descriptive statistics. Microsoft Excel 2016 (Microsoft Corp, Redmond, WA, USA) was used to collate and analyse numerical data. Median and range values were obtained for the dataset.

## Results

### Response rate

**Figure 1:**
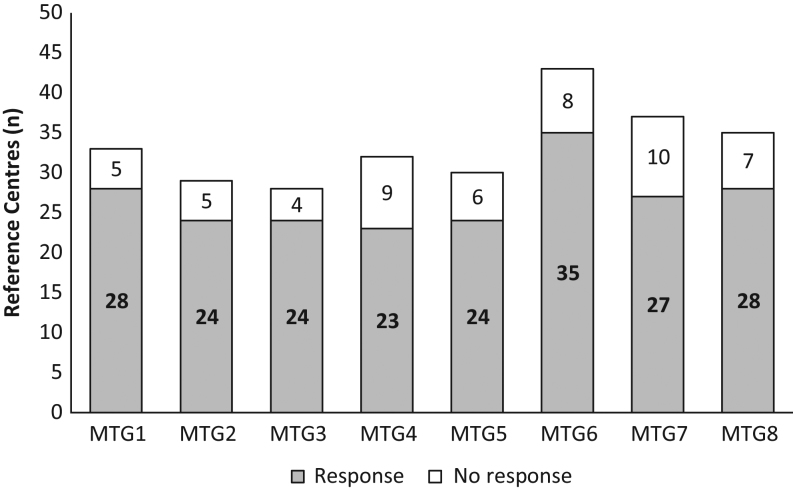
Response rates for reference centres (RC) categorised according to the Main Thematic Groups (MTG). Of the 71 RC, 58 (82%) responded to the survey. The mean response rate across 8 themes was 80%. MTG1, adrenal; MTG2, calcium and phosphate; MTG3, glucose and insulin; MTG4, endocrine tumours; MTG5, growth and obesity; MTG6, pituitary; MTG7, sex development; MTG8, thyroid.

A response rate of 82% (58/71 RC) was obtained. Twenty-five (35%) RCs were involved in six or more themes and 26 (37%) RCs were involved in one theme only. The average response rate across all eight themes was 80% (Fig. 1).

### Adrenal registries

**Figure 2:**
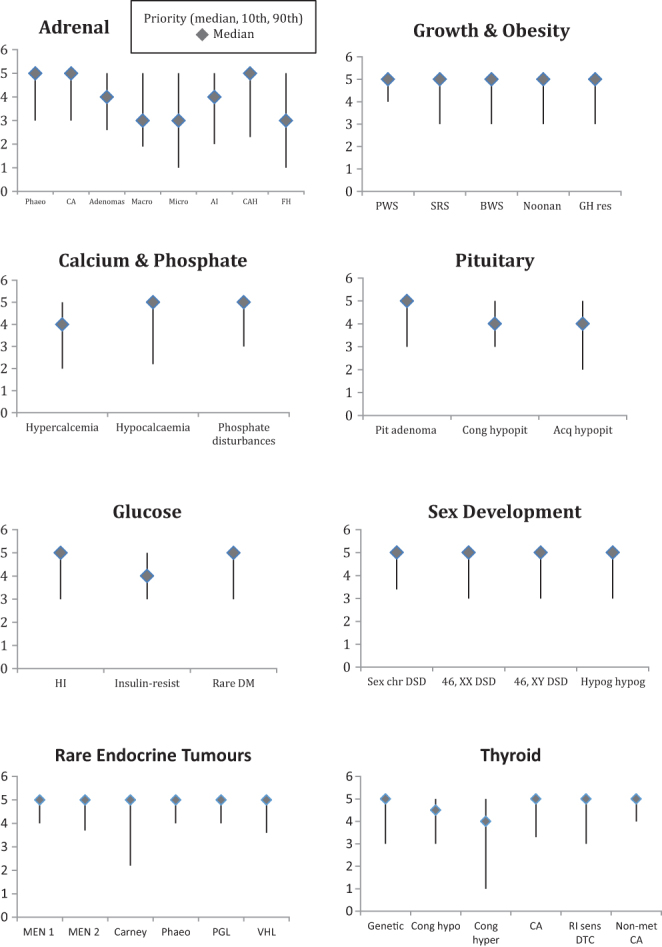
Views on future priorities categorised according to the Main Thematic Groups (MTG). A score of 5 (highest priority for need of a registry) was attributed to all conditions within MTG4, MTG5 and MTG7. MTG1, adrenal; MTG2, calcium and phosphate; MTG3, glucose and insulin; MTG4, endocrine tumours; MTG5, growth and obesity; MTG6, pituitary; MTG7, sex development; MTG8, thyroid.

Of the 33 centres surveyed within the adrenal theme, awareness of an international registry was 68% for adrenocortical tumours, 57% for phaeochromocytoma, 54% for congenital adrenal hyperplasia (CAH), 46% for bilateral macro- and micro-nodular adrenal hyperplasia and 29% for adrenal insufficiency (Table 1). International registry participation rates were 39, 30, 18, 21 and 6% respectively. A median priority score for future registry development of 5 was attributed to adrenocortical tumours, phaeochromocytoma and CAH (Fig. 2).

**Table 1:** Survey of Endo-ERN reference centres (RC) showing extent of awareness and participation in rare disease registries for conditions included in Endo-ERN within the 8 broad main thematic groups (MTG). Data is presented as *n* or *n* (%)

Endo-ERN MTGs and conditions surveyed	RC surveyed	RC responded	Awareness of registries			Participation in registries		
			International	National	Local	International	National	Local
MTG1. Adrenal	33	28 (85)						
Phaeochromocytoma			16 (57)	7 (25)	16 (57)	10 (36)	6 (21)	14 (50)
Adrenocortical carcinoma			20 (71)	15 (54)	15 (54)	13 (46)	6 (21)	15 (54)
Cortisol producing adenomas			19 (68)	8 (29)	12 (43)	12 (43)	8 (29)	12 (43)
Bilateral macronodular hyperplasia			13 (46)	4 (14)	11 (39)	8 (29)	4 (14)	11 (39)
Bilateral micronodular hyperplasia			13 (46)	3 (10)	10 (36)	7 (25)	3 (11)	10 (36)
(Primary) adrenal insufficiency			8 (29)	5 (18)	13 (46)	2 (7)	5 (18)	13 (46)
Congenital adrenal hyperplasia			15 (54)	15 (54)	17 (61)	6 (21)	15 (54)	17 (61)
Familial hyperaldosteronism			6 (21)	4 (14)	8 (29)	3 (11)	4 (14)	6 (21)
MTG2. Calcium and phosphate	29	24 (83)						
Hypercalcaemia			3 (13)	4 (17)	11 (46)	2 (8)	4 (17)	11 (46)
Hypocalcaemia			1 (4)	2 (8)	13 (54)	0 (0)	2 (8)	13 (54)
Phosphate disturbances			4 (17)	4 (17)	15 (63)	2 (8)	2 (8)	15 (63)
MTG3. Glucose amd insulin	29	24 (83)						
Hyperinsulinism			5 (21)	9 (38)	10 (42)	3 (12)	6 (24)	9 (38)
Insulin-resistance syndrome			12 (50)	8 (33)	6 (24)	9 (38)	8 (33)	6 (24)
Rare diabetes mellitus			18 (75)	16 (67)	12 (50)	14 (56)	16 (67)	12 (50)
MTG4. Endocrine tumours	31	23 (74)						
MEN type 1			6 (26)	8 (35)	16 (70)	2 (9)	8 (35)	16 (70)
MEN type 2			5 (22)	4 (17)	15 (65)	1 (4)	4 (17)	15 (65)
Carney complex			1 (4)	1 (4)	8 (35)	0 (0)	1 (4)	7 (30)
Hereditary phaeochromocytoma–paraganglioma			8 (35)	7 (30)	14 (61)	4 (17)	7 (30)	14 (61)
Von Hippel Lindau syndrome			6 (26)	4 (17)	15 (65)	3 (13)	4 (17)	15 65)
MTG5. Growth and obesity	30	24 (80)						
Prader–Willi and Prader–Willi-like syndrome			5 (21)	16 (67)	19 (79)	3 (13)	14 (58)	18 (75)
Silver Russell syndrome			3 (13)	9 (38)	10 (42)	1 (4)	7 (29)	9 (38)
Beckwith Wiedemann syndrome			1 (4)	3 (13)	6 (24)	1 (4)	3 (12)	5 (21)
Noonan syndrome			2 8)	9 (38)	10 (42)	1 (4)	9 (38)	9 (38)
GH resistance syndromes			3 (13)	5 (21)	9 (38)	1 (4)	5 (21)	9 (38)
MTG6. Pituitary	43	35 (81)						
Pituitary adenoma			14 (43)	17 (49)	24 (69)	10 (29)	17 (49)	24 (69)
Congenital hypopituitarism			7 (20)	12 (34)	19 (54)	5 (14)	11 (31)	19 (54)
Acquired hypopituitarism			9 (25)	13 (37)	20 (57)	6 (17)	13 (37)	20 (57)
MTG7. Sex development	37	27 (73)						
Sex chromosome DSD			18 (67)	12 (44)	18 (67)	13 (48)	10 (37)	18 (67)
46, XX DSD			19 (70)	12 (44)	17 (63)	14 (52)	10 (37)	17 (63)
46, XY DSD			19 (70)	13 (48)	18 (67)	14 (52)	11 (41)	18 (67)
Congenital hypogonadotrophic hypogonadism			15 (56)	10 (37)	16 (59)	9 (33)	10 (37)	16 (59)
MTG8. Thyroid	35	28 (80)						
Thyroid signalling disorders			6 (21)	4 (14)	8 (29)	2 (7)	4 (14)	7 (25)
Congenital hypothyroidism			2 (7)	18 (64)	12 (43)	0 (0)	15 (54)	11 (39)
Congenital hyperthyroidism			1 (4)	1 (4)	4 (14)	0 (0)	1 (4)	3 (11)
Non-metastatic thyroid macro-carcinoma			5 (18)	15 (54)	16 (57)	0 (0)	15 (54)	16 (57)
Radioiodine sensitive differentiated thyroid cancer			3 (11)	8 (29)	12 (43)	0 (0)	8 29)	12 (43)
Non-metastatic medullary thyroid carcinoma			4 (14)	11 (39)	14 (50)	0 (0)	11 (39)	14 (50)

### Registries for disorders of calcium and phosphate homeostasis

Of the 29 centres within the calcium/phosphate theme, international registry awareness was 17% for disorders of phosphate disturbances, 13% for hypercalcaemia and 4% for hypocalcaemia; participation was 8, 8 and 0%, respectively. Awareness and participation in local registries was higher for all conditions (Table 1). Hypocalcaemia and hypophosphataemia were given a median priority score of 5 (Fig. 2).

### Registries for genetic disorders of glucose and insulin homeostasis

Of the 29 centres within the glucose theme, participants reported the highest rates of awareness and participation in an international registry for rare diabetes (75 and 56%, respectively) (Table 1). Median priority scores of 5 were attributed to rare diabetes and congenital hyperinsulinism (Fig. 2).

### Registries for genetic endocrine tumour syndromes

Of the 31 centres reporting on the rare genetic tumour theme, 26% reported an international registry awareness for MEN1 and 9% reported participation (Table 1). Seventy percent reported awareness and participation in local registries for MEN1. All conditions were rated as 5 on priority scoring (Fig. 2).

### Registries for growth and genetic obesity syndromes

Of the 30 centres within the growth theme, local and national registry awareness and participation for all conditions was greater than that for international registries. International registry awareness was highest for Prader–Willi syndrome at 17% with a participation level of 10% (Table 1). Participation in international registries for Silver–Russell syndrome, Beckwith–Wiedemann syndrome and Noonan syndrome was 3%. All conditions within the growth theme were given a median priority score of 5 (Fig. 2).

### Pituitary registries

Of the 43 centres within the pituitary theme, awareness and participation in an international registry was greatest for pituitary adenomas, with 43% awareness and 29% participation (Table 1). Pituitary adenoma was the only condition given a median priority score of 5 (Fig. 2).

### Registries for sex development and maturation

Of the 37 centres within the sex development theme, 70% reported an awareness and 52% reported participation in international registries for 46, XX and 46, XY disorders or differences of sex development (DSD) (Table 1). Despite greater awareness of international registries, rates of participation in local registries were higher than those for international registries for all DSD (49% vs 39%). All conditions within the sex development group were given a median priority score of 5 (Fig. 2).

### Thyroid registries

Of the 35 centres within the thyroid theme, international registry awareness was greatest for thyroid signalling disorders (21%), however, participation was only 7% (Table 1). Out of the six conditions, four (thyroid signalling disorders, thyroid carcinoma, radioiodine sensitive differentiated thyroid carcinoma (RI sensitive DTC), non-metastatic medullary thyroid disease) were rated as 5 (Fig. 2).

### Comparison of Endo-ERN, Orphanet and RD-Connect

**Figure 3:**
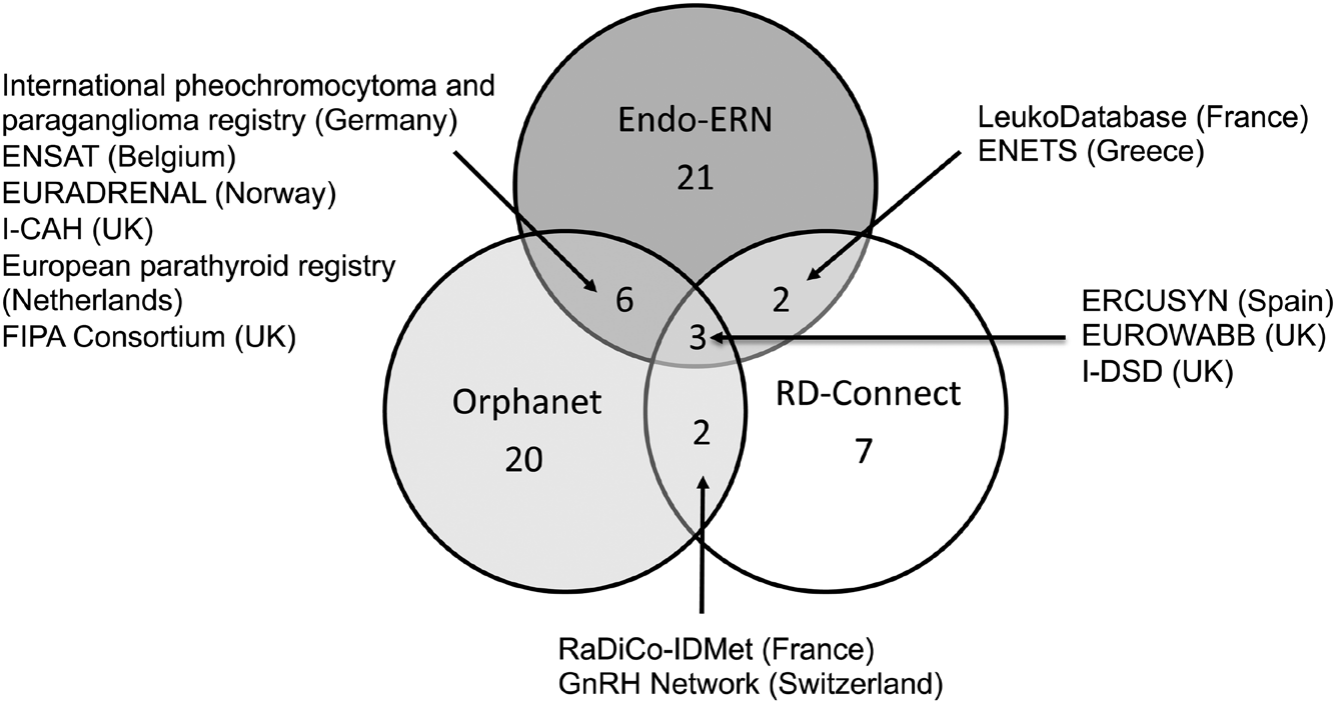
International registries for rare endocrine conditions. 33 registries were identified via Endo-ERN members, Orphanet and RD-Connect.

For endocrine conditions within the main thematic groups of Endo-ERN, 33 registries were identified through the above survey of Endo-ERN and the search of the Orphanet and RD-Connect databases (Table 2). Endo-ERN members identified 21 registries, Orphanet identified 20 and RD-Connect identified 7. Of the 27 registries identified via Orphanet or RD-Connect, Endo-ERN members reported 11 (41%) (Fig. 3). Amongst the international registries identified, several existed for discrete groups of conditions including Adrenal, Genetic Disorders of Glucose and Insulin Homeostasis and Growth and Obesity Syndromes. Three international registries were identified by Endo-ERN, Orphanet and RD-Connect, and these included ERCUSYN (European Registry on Cushing’s Syndrome, www.lb.de/ercusyn), EUROWABB (EU Rare Diseases Registry for Wolfram Syndrome, Alstrom Syndrome, Bardet–Biedl Syndrome and Other Rare Diabetes Syndromes, www.euro-wabb.org) and I-DSD (International Registry for Disorders of Sex Development, www.i-dsd.org) which also includes CAH. Of the six industry-led registries that were identified by Endo-ERN RC, none were identified in Orphanet and RD-Connect (Table 2), whereas, of the 27 non-commercial registries, all were identified through Orphanet, RD-Connect or both.

**Table 2:** International rare disease registries with a coordinating centre within Europe for conditions within Endo-ERN categorised according to the main thematic group (MTG) and their level of awareness within Endo-ERN, Orphanet and RD-Connect.

International rare disease registry (co-ordinating centre within Europe)	Endo ERN MTGS (1–8)	Endo-ERN conditions (orphacode)	Orphanet	RD-Connect	Awareness-Endo-ERN
International Pheochromocytoma and Paraganglioma Registry (Freiburg, Germany)	1, 4	Sporadic phaeochromocytoma–paraganglioma (PCC/PGL) (ORPHA276624); hereditary PCC/PGL (ORPHA29072); Von Hippel Lindau syndrome (ORPHA892)	+	–	+
ENSAT: European Network for the Study of Adrenal Tumours (Brussels, Belgium)	1, 4	Sporadic phaeochromocytoma–paraganglioma (PCC/PGL) (ORPHA276624); adrenocortical carcinoma (ORPHA1501); MEN type 1 (ORPHA652); MEN type 2 (ORPHA653); carney complex (ORPHA1359); hereditary PCC/PGL (ORPHA29072); Von Hippel Lindau syndrome (ORPHA892)	+	–	+
EU-AIR: European Adrenal Insufficiency Registry (Shire, Zug, Switzerland)	1	(Primary) adrenal insufficiency (ORPHA101959)	–	–	+
EURADRENAL: European Patient Registry on Autoimmune Adrenal Failure (Bergen, Norway)	1	(Primary) adrenal insufficiency (ORPHA101959)	+	–	+
I-CAH: International Registry for Congenital Adrenal Hyperplasia (Glasgow,UK)	1, 7	Congenital adrenal hyperplasia (ORPHA418); 46, XX DSD (ORPHA2982)	+	–	+
ERCUSYN: European Registry on Cushing’s Syndrome (Barcelona, Spain)	1, 4, 6	Adrenocortical carcinoma (ORPHA1501); cortisol producing adenomas (ORPHA443287); MEN type 1 (ORPHA652); MEN type 2 (ORPHA653); carney complex (ORPHA1359); pituitary adenoma (ORPHA99408)	+	+	+
ESPN/ERA-EDTA Registry: European Registry for Children on Renal Replacement Therapy (Amsterdam, Netherlands)	1, 4, 8	Adrenocortical carcinoma (ORPHA1501); hereditary PCC/PGL (ORPHA29072); Von Hippel Lindau syndrome (ORPHA892); congenital hyperthyroidism (ORPHA424)	+	–	–
X-ALD: X-linked Adrenoleukodystrophy Database (Amsterdam, Netherlands)	1	(Primary) adrenal insufficiency (ORPHA101959)	+	–	–
European LeukoDatabase (LeukoDB) (Paris, France)	1	(Primary) adrenal insufficiency (ORPHA101959)	–	+	+
European Parathyroid Tumour Registry (Leiden, Netherlands)	2	Rare forms of hyperparathyroidism including parathyroid cancer and FHH (ORPHA181408)	+	–	+
EUROGLYCANET-International Patient Registry and Cohort for Congenital Disorders of Glycosylation (Leuven, Belgium)	2	Hyperphosphatemia (ORPHA306661)	+	–	–
XLH (X-linked Hypophosphataemia) Registry (Kyowa Kirin, Galashiels, UK)	2	Hypophosphatemia (ORPHA 89937)	–	–	+
UK10K_RARE_SIR-The Severe Insulin Resistance (SIR) Variant Database (HInxton, UK)	3, 5	Insulin-resistance syndrome (ORPHA181368); rare diabetes mellitus (ORPHA101952); rare genetic obesity (ORPHA77828)	+	–	–
EUROWABB: An EU Rare Diseases Registry for Wolfram Syndrome, Alstrom Syndrome, Bardet-Biedl Syndrome and Other Rare Diabetes Syndromes (Birmingham, UK)	3, 5	Rare diabetes mellitus (ORPHA101952); rare genetic obesity (ORPHA77828)	+	+	+
SWEET: ‘Better control in Pediatric and Adolescent diabetes: Working to create centers of reference’ (Hannover, Germany)	3	Rare diabetes mellitus (ORPHA101952)	–	–	+
EcLip: European Consortium of Lipodystrophies (Ulm, Germany)	3	Rare diabetes mellitus (ORPHA101952)	–	–	+
ENETS: European Neuroendocrine Tumour Society (Athens, Greece)	4	Hereditary PCC/PGL (ORPHA29072)	–	+	+
Cooperative European Paediatric Renal Transplant Initiative registry (Heidelberg, Germany)	4, 6	Von Hippel Lindau syndrome (ORPHA892); congenital hyperthyroidism (ORPHA424)	+	–	–
MD-NET: Muscle Tissue Culture Collection (MTCC) (EuroBioBank partner) (Munchen, Germany)	5	Prader–Willi and Prader–Willi-like syndrome (ORPHA739); rare genetic obesity (ORPHA77828)	+	–	–
European Prader–Willi Syndrome Database (Cambridge, UK)	5	Prader–Willi and Prader–Willi-like syndrome (ORPHA739); rare genetic obesity (ORPHA77828)	+	–	–
RaDiCo-IDMet-French and European Cohort in Imprinting Disorders and Metabolism Future (Paris, France)	2, 3, 5	Hypoparathyroidism (ORPHA181405); rare diabetes mellitus (ORPHA101952); Prader–Willi and Prader–Willi-like syndrome (ORPHA739); Silver Russell syndrome (ORPHA813); Beckwith Wiedemann syndrome (ORPHA116); rare genetic obesity (ORPHA77828)	+	+	–
IGFD Registry: The Increlex Growth Forum Database (Ipsen, Paris, France)	5	GH resistance syndromes (ORPHA633)	–	–	+
Liège Acromegaly Survey (LAS) Database (Lèige, Belgium)	5, 6	Overgrowth syndrome (ORPHA93460); pituitary adenoma (ORPHA99408)	–	–	+
KIMS (Pfizer International Metabolic Study) – Adults with GH Deficiency (Pfizer, Stockholm, Sweden)	5	GH resistance syndromes (ORPHA633); congenital hypopituitarism (ORPHA95494); acquired hypopituitarism (ORPHA95502)	–	–	+
KIGS (Pfizer International Growth Study) – (Pfizer, Stockholm, Sweden)	5, 6, 7	Prader–Willi and Prader–Willi-like syndrome (ORPHA739); Silver Russell syndrome (ORPHA813); congenital hypopituitarism (ORPHA95494); acquired hypopituitarism (ORPHA95502); sex chromosome DSD (ORPHA325546)	–	–	+
ACROSTUDY (International Somavert Database) (Pfizer, Stockholm, Sweden)	5, 6	Overgrowth syndrome (ORPHA93460); pituitary adenoma (ORPHA99408)	–	–	+
Nordinet International Outcome Study (Novo Nordisk, Bagsvaerd, Denmark)	5,7	Prader–Willi and Prader–Willi-like syndrome (ORPHA739); Noonan syndrome (ORPHA648); GH resistance syndromes (ORPHA633); sex chromosome DSD (ORPHA325546)	–	–	+
DYSCERNE’s Dysmorphology Diagnostic System (DDS) (Manchester, UK)	5	Silver Russell syndrome (ORPHA813)	+	–	–
Global Familial Isolated Pituitary Adenoma (FIPA) consortium (London, UK)	6	Pituitary adenoma (ORPHA99408)	+	–	+
COST Action BM1105 Patient Registry-GnRH Network (Lausanne, Switzerland)	6 , 7	Congenital hypopituitarism (ORPHA95494); isolated congenital anosmic hypogonadotrophic hypogonadism (ORPHA478); isolated congenital normosmic hypogonadotrophic hypogonadism (ORPHA432)	+	+	–
I-DSD: International Registry for Disorders of Sex Development (Glasgow, UK)	7	Sex chromosome DSD (ORPHA325546); 46, XX DSD (ORPHA2982); 46, XY DSD (ORPHA98085)	+	+	+
UK10K_RARE_THYROID-Congenital Hypothyroidism Variant Database (Cambridge, UK)	8	Thyroid signalling disorders (ORPHA183631); congenital hypothyroidism (ORPHA442)	+	–	–
MCT8 Registry. International registry of rare thyroid disorders (Rotterdam, the Netherlands)	8	Thyroid signalling disorders (ORPHA183631)	–	–	+

MTG1, adrenal; MTG2, calcium and phosphate; MTG3, glucose and insulin; MTG4, endocrine tumours; MTG5, growth and obesity; MTG6, pituitary; MTG7, sex development; MTG8, thyroid.

## Discussion

This contemporary survey of registries has clearly revealed that whilst there are several registries for a range of rare endocrine conditions, there are also several gaps in coverage of conditions. In addition, there are gaps in the level of awareness and use as well as the international profile of existing international registries.

Clear patterns were observed for awareness and participation of international registries. For conditions including disorders of calcium and phosphate, endocrine tumour syndromes, growth syndromes and pituitary and thyroid disease, there was greater awareness of local or national registries rather than international registries. On the other hand, for other groups of conditions such as adrenal disorders, insulin resistance syndrome, rare diabetes mellitus and DSD, there was a high level of awareness and participation in international registries.

The increased awareness of international registries amongst the Endo-ERN participants for particular subgroups of conditions may be attributed to the presence of already established secure Web-based registries such as those listed within Orphanet and RD-Connect and/or to large international collaboration of RC within a given theme prior to the establishment of Endo-ERN, for example, the glucose, adrenal and DSD themes. The SWEET registry is an example of a registry that was initially developed to cover type 1 diabetes mellitus; however, in doing so, it has also become a powerful resource for rare forms of diabetes (8). Although the use of international, cross-border registries may be primarily linked to the availability and awareness of registries, many RC leads were not aware of international registries or did not use these registries despite being aware of them. Reasons for not participating in registries require further exploration, however, previous studies have reported that these may include failure to obtain consent and lack of time or personnel (9). It is also possible that an absence of transparent quality indicators that can be used to assess registries (10) may also play a role. However, the current study suggests that lack of awareness of a registry may be a very important factor. Conditions for which a registry was considered a high priority were often those for which international registries already existed. There may be several explanations for this finding. Firstly, it may simply reflect the respondent’s lack of awareness of the existing registries. Alternatively, it may reflect the respondent’s belief that the existing registry needs to remain high priority or the respondent’s view that another new registry needs to be developed.

Databases such as Orphanet and RD-Connect have been developed to facilitate greater awareness of registries for rare conditions and may also pave the way towards improving the quality of registries. However, it is important to note that current databases of rare disease registries such as Orphanet and RD-Connect did not have a record of several international registries that are currently led from Europe. This was noted particularly for rare disease registries led by industry. Given that rare disease registries have to enter their details on these databases, the discordance suggests a mutual lack of awareness between these databases and the rare disease registries of the respective platforms. It is possible that registries led by industry have been designed specifically for post-marketing surveillance of specific drugs for a fixed period with limited applicability to the wider community and long-term sustainability may not be a priority, and this may explain their lack of visibility on Orphanet and RD-Connect. On the other hand, these registries are generally well resourced with a clear objective, and it is possible that the data within them are of high quality. In the field of rare conditions, greater partnership with industry is generally supported by all stakeholders (European Medicines Agency Report on the Patient Registries Workshop (28 October 2016) http://www.ema.europa.eu/docs/en_GB/document_library/Report/2017/02/WC500221618.pdf; last accessed September 13 2018) and is necessary for effective use of limited resources.

Whilst the current survey shows that many centres are collecting data in local registries, participation in international registries is often considered to be the gold standard for improving the quality of research, improving patient care and the external validity of registries for rare conditions (11, 12, 13). However, one of the most challenging aspects of developing an international detailed disease registry is the requirement of a common dataset based on a standardised data entry (10). It is highly likely that these separate registries will have several fields that will be common to all. Thus, the existence of a group of local and national registries should be viewed positively as these registries can pave the way towards the development of a common dataset for an international registry. Another hurdle in the development of multinational registries has long been the existence of country-specific ethical regulations for the development and use of such registries. The recently issued General Data Protection Regulation (GDPR) by the EU provides a welcome opportunity to overcome these problems (14).

With the recent development of Endo-ERN, consideration should also be given to effective collation of available resources and development of a virtual environment that serves the objectives of this ERN and one that acts as a model for the wider endocrine community as well as other rare disease networks (15). The European Registries for Rare Endocrine Conditions (EuRRECa) [eurreca.net] is a new project that aims to address gaps raised in the above survey by developing a core registry and an e-reporting programme for all rare endocrine conditions that are covered within Endo-ERN. EuRRECa will not only increase the awareness of registries amongst patients and professionals by facilitating interaction between existing and new disease registries within Endo-ERN, it will also assess the quality of existing registries using agreed guidance (11, 12) and signpost the endocrine community to these registries. By partnering with the European Society for Endocrinology and the European Society for Paediatric Endocrinology, the project will offer its resources to a wider group of health care professionals beyond Endo-ERN. It is also anticipated that the project will act as a bridge between the endocrine community and global initiatives that seek to create an inventory of existing registries.

In summary, whilst there is a clear need to develop new detailed disease registries, there is also a need to improve the awareness and signposting of existing registries. A common platform that can be used by the whole endocrine community and which directs the user to high-quality detailed disease registries has the potential to achieve this objective.

## Declaration of interest

Olaf Dekkers is an editor of the European Journal of Endocrinology. There are no other conflicts of interest that could be perceived as prejudicing the impartiality of this study.

## Funding

This publication is part of the project ‘777215/EuRRECa’ which has received funding from the European Union’s Health Programme (2014–2020).
